# Healthcare Costs for Treating Relapsing Multiple Sclerosis and the Risk of Progression: A Retrospective Italian Cohort Study from 2001 to 2015

**DOI:** 10.1371/journal.pone.0169489

**Published:** 2017-01-05

**Authors:** Marcello Moccia, Raffaele Palladino, Roberta Lanzillo, Antonio Carotenuto, Cinzia Valeria Russo, Maria Triassi, Vincenzo Brescia Morra

**Affiliations:** 1 Multiple Sclerosis Clinical Care and Research Centre, Department of Neurosciences, Reproductive Sciences and Odontostomatology, Federico II University, Naples, Italy; 2 Department of Primary Care and Public Health, Imperial College, London, United Kingdom; 3 Department of Public Health, Federico II University, Naples, Italy; Washington University, UNITED STATES

## Abstract

**Background:**

Disease modifying treatments (DMTs) are the main responsible for direct medical costs in multiple sclerosis (MS). The current investigation aims at evaluating possible associations between healthcare costs for treating relapsing remitting MS (RRMS) and disease evolution.

**Methods:**

The present cohort study retrospectively included 544 newly diagnosed RRMS patients, prospectively followed up for 10.1±3.3 years. Costs for DMT administration and management were calculated for each year of observation. Following clinical endpoints were recorded: time to first relapse, 1-point EDSS progression, reaching of EDSS 4.0, reaching of EDSS 6.0, and conversion to secondary progressive MS (SP). Covariates for statistical analyses were age, gender, disease duration and EDSS at diagnosis.

**Results:**

At time varying Cox regression models, 10% increase in annual healthcare costs was associated with 1.1% reduction in 1-point EDSS progression (HR = 0.897; p = 0.018), with 0.7% reduction in reaching EDSS 6.0 (HR = 0.925; p = 0.030), and with 1.0% reduction in SP conversion (HR = 0.902; p = 0.006).

**Conclusion:**

Higher healthcare costs for treating MS have been associated with a milder disease evolution after 10 years, with possible reduction of long-term non-medical direct and indirect costs.

## Introduction

Multiple Sclerosis (MS) is a chronic inflammatory disease of the central nervous system causing neurodegeneration, axonal injury and demyelination, and is responsible for significant physical disability and social impact. Direct and indirect costs associated with MS are high, due to its chronic management and to early onset in economically productive years [1,2].

The introduction of Disease Modifying Treatments (DMTs) during the last 20 years has been changing the natural history of MS. Clinical trials support the early initiation of chronic DMTs in order to avoid short term disease activity (i.e. relapses), and to reduce or, ideally, to prevent long term disability accrual [3–6]. However, few studies have been conducted in the long term and, apparently, the use of DMTs might be responsible for delaying the reaching of milestones of disability progression [4,7]. Notwithstanding this, DMTs are nowadays the main responsible for direct healthcare costs in MS, and are expected to have a further expansion among overall expenses as a consequence of the introduction of newer and more effective drugs characterized by high healthcare costs for administration and management [8–11].

Soaring healthcare costs for DMTs have received much attention in health policy contexts, with some studies estimating DMT costs being beyond the health care system tolerance, whereas others below the threshold [11,12]. In particular, there are no investigations on whether the long-term economic burden for administration and management of DMTs is associated with MS clinical evolution [10,13]. Therefore, the present retrospective cohort study aims to explore the relationships between the expenditure for MS treatments, and the risk of relapses and of disability progression during a 10-year observation period.

## Methods

### Study design

The present observational cohort study is a retrospective analysis of prospectively collected data, recorded in the database of the MS Clinical Care and Research Centre of the Federico II University Hospital of Naples, Italy.

In compliance with current Italian applicable laws and regulations, considering that all clinical assessments were part of clinical practice in a University setting and that the retrospective analysis included anonymized data, specific ethics approval was not required. All subjects signed the general informed consent form, authorizing the use of personal data for research purposes. The study was performed in accordance with the good clinical practice and the Declaration of Helsinki.

### Population

Inclusion criteria were: 1) new diagnosis of RRMS from January 2001 to January 2010 [14,15]; 2) indication for the use of DMTs; 3) presence of at least 5 year clinical follow-up.

Exclusion criteria were: 1) progressive forms of MS at baseline [15]; 2) age at diagnosis <18 years; 3) previous use of DMTs; 4) participation in clinical trials and/or observational studies for DMTs during the study period; 5) pregnancy during the study period; 6) incomplete clinical records.

Due to the observational nature of the study, DMTs were possibly discontinued or changed during the study period in accordance with the local regulatory indications for clinical practice (Italian and European Medicines Agencies) [16,17]. In particular, at the time of data extraction (December 2015), Dimethyl Fumarate, Glatiramer acetate, Interferon beta-1-a, Interferon beta-1-b, and Teriflunomide were approved for being prescribed in patients with a diagnosis of RRMS, whereas Alemtuzumab, Fingolimod, and Natalizumab were limited to patients with highly active RRMS. DMTs for highly active RRMS were those with the highest costs for administration and management [18].

### Economic variables

Economic resources for the present study included the healthcare costs for the DMTs, for the staff involved in DMT administration (either for training the patient and his/her caregiver in self-administration, or for the inpatient administration procedures), for neurological visits, for other specialist visits related to DMT safety procedures (i.e. ophthalmology), for MRI procedures, for laboratory exams, for psychological and neuropsychological evaluations, performed in accordance with current guidelines and clinical practice [16,17,19,20]. These procedures were selected for the analyses since they were directly monitored by the MS Centre and recorded in the clinical database.

Healthcare costs were inflated to the most recent values, obtained from the National Drug Formulary for DMT costs (Italian Drug Agency), and from the National Tariffs for Healthcare of the Italian National Health System for resource utilization costs (Italian Ministry of Health), as previously performed in similar studies on Italian MS populations, in order to avoid variations in price per unit of service through different years [18,21].

Healthcare costs were referred to each year of observation (annual healthcare costs), in order to obtain annual healthcare costs before study endpoints were reached. Overall annual healthcare costs were calculated for each patient (sum of healthcare costs from the whole study period/years of study). Healthcare costs were included in the statistical models as continuous variables in order to avoid information loss [22]. For statistical purposes, in order to reduce skewness of the data, all healthcare costs underwent logarithmic transformation (log).

### Clinical outcomes

During the whole study period, MS subjects attended the MS Centre for visits which were scheduled according to the clinical practice for the follow-up of their disease and treatment, or for the occurrence of a clinical relapse, and were evaluated for 5 different clinical endpoints that have previously been demonstrated as being milestones of MS evolution [23]:

- Occurrence of clinical relapse: relapses occurring during the study period were recorded; the time occurring from MS diagnosis to the first relapse was calculated (time to first relapse); then, the overall number of relapses was reported on an annual basis (annualized relapse rate -ARR-); relapsing patients presented a range of motor/sensory symptoms and met commonly used standards for relapse as determined by clinical neurologists [14];
- 1-point Expanded Disability Status Scale (EDSS) progression (confirmed after 12 months, and independent from the occurrence of relapses): the cohort was categorized according to the 1-point EDSS progression or not, and the time from the diagnosis to the 1-point EDSS progression was calculated (time to 1-point EDSS progression);
- Reaching of EDSS 4.0 (confirmed after 12 months): the cohort was categorized according to the reaching of EDSS 4.0 or not, and the time from the diagnosis to the reaching of EDSS 4.0 was calculated (time to EDSS 4.0);
- Reaching of EDSS 6.0 (confirmed after 12 months): the cohort was categorized according to the reaching of EDSS 6.0 or not, and the time from the diagnosis to the reaching of EDSS 6.0 was calculated (time to EDSS 6.0);
- Transition from RR to SP course: the cohort was categorized according to the conversion to SP or not, and the time from the diagnosis to the SP conversion was calculated (time to SP); MS was considered SP when a progressive accumulation of disability occurred after an initial relapsing course, and was associated with a worsening of the same functional system, independently from relapse activity [15,24].

Endpoints of disability progression (1-point EDSS progression, reaching of EDSS 4.0, reaching of EDSS 6.0, and SP conversion) were chosen because clinically important and unlikely to remit once sustained [20,23,25]. The observation period was extended to 12 months in order not to wrongly estimate disability accrual [26]. Clinical evaluations were performed by trained neurologists qualified for EDSS assessment.

### Covariates

Covariates included in the models were recorded at the time of diagnosis, and included demographic characteristics (age, gender), disease duration (time occurring from the reported onset of the first clinical symptom to the diagnosis), and the EDSS [27], which have previously been associated with the 10-year direct costs for treating MS [18].

### Statistics

Means and proportions of demographic features, clinical findings and healthcare costs were calculated for the MS population. All healthcare costs underwent logarithmic transformation before being included in the statistical models as continuous variables.

Time varying Cox regression models were performed to evaluate the associations between the annual healthcare costs before the specific study endpoint was reached, and the hazard ratios (HR) of relapse occurrence (time to the first relapse), of 1-point EDSS progression, of reaching of EDSS 4.0, of reaching of EDSS 6.0, and of conversion to SP; 95% confidence interval (95%CI) were subsequently calculated. Models were adjusted for age, gender, disease duration and baseline EDSS. In order to improve data presentation, results from the Cox regression models were discussed considering the effect of a 10% increase in the healthcare costs using the following formula: $\hat{\beta }$*ln(1.10).

Poisson regression models were performed to evaluate associations between overall annual healthcare costs and ARR; coefficients (Coef) and 95%CI were subsequently calculated. Models were adjusted for age, gender, disease duration, baseline EDSS, and follow-up duration.

Covariates were preliminary studied in relation to the overall annual healthcare costs by using t-test for categorical variables, linear regression analysis for continuous variables, and ordinal regression analysis for ordinal variables.

For data presentation in Kaplan-Meier curves, healthcare costs have been categorized on the median value (10801.92€ per year). Patients treated with either Fingolimod or Natalizumab during at least a period of the observation time were more likely to be part of the group with healthcare costs higher than the median value (p<0.001).

Results have been considered statistically significant if p<0.05. Stata 12.0 has been used for data processing and analysis.

## Results

**Fig 1:**
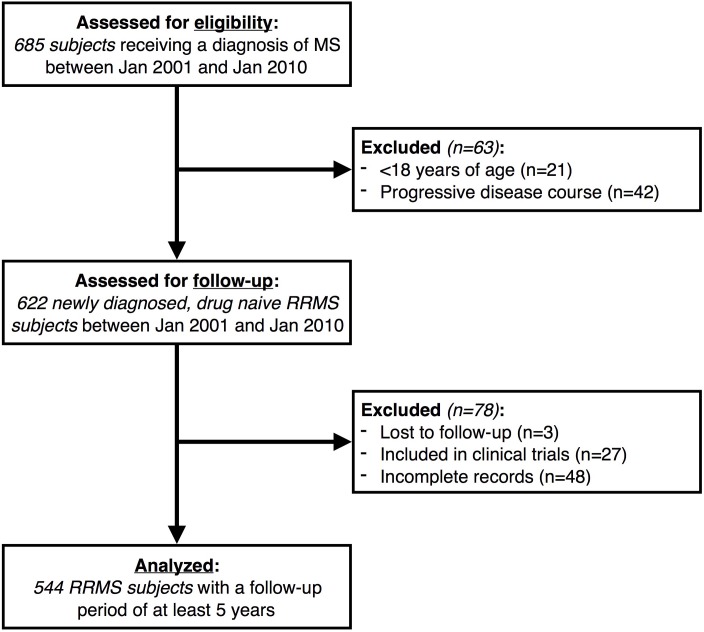
Patient disposition flow diagram. Patient disposition flow diagram showing patients included, excluded or lost-to-follow-up within the study cohort. MS: Multiple Sclerosis; RRMS: Relapsing-Remitting Multiple Sclerosis.

Among 685 eligible subjects, the present study included 544 newly diagnosed, drug naïve RRMS patients, who were followed-up for 10.2±3.4 years. Details of subjects assessed for eligibility and excluded or included in the present study, are reported in Fig 1.

Demographic features, clinical findings and healthcare costs of the MS cohort are reported in Table 1. Additional clinical and economic characteristics of the present population have been fully described elsewhere [18].

**Table 1:** Demographic features, clinical findings and healthcare costs. Demographic features, clinical findings and annual healthcare costs for DMT administration and management.

	*MS population (n = 544)*
**Age**, *average years±SD*	33.7±8.7
**Gender**, *number of females (percent)*	345 (63.5%)
**Disease duration at diagnosis**, *average years±SD*	3.1±3.3
**EDSS at diagnosis**, *median (IQR)*	2 (1.5–2.5)
**Observation period**, *average years±SD*	10.2±3.4
**Overall annual healthcare costs**, *€±SD*	11785.35±2718.76
**Relapse occurrence**, *number (percent)*	415 (76.2%)
**Time to the first relapse**, *average years±SD*	2.7±2.5
**1-point EDSS progression**, *number (percent)*	448 (82.3%)
**Time to 1-point EDSS progression**, *average years±SD*	4.5±4.0
**Reaching of EDSS 4.0**, *number (percent)*	256 (47.0%)
**Time to EDSS 4.0**, *average years±SD*	7.0±3.7
**Reaching of EDSS 6.0**, *number (percent)*	59 (10.8%)
**Time to EDSS 6.0**, *average years±SD*	10.3±3.6
**Conversion to SP**, *number (percent)*	102 (18.7%)
**Time to SP conversion**, *average years±SD*	8.6±3.3

(MS: Multiple Sclerosis; EDSS: Expanded Disability Status Scale; ARR: Annualized Relapse Rate; SP: Secondary Progressive; DMTs: Disease Modifying Treatments).

In agreement with the indications of the Italian regulatory agency, the first prescribed DMT was discontinued and possibly changed in 291 patients (53.4%), which underwent 2.2±1.1 different DMTs during the 10-year study period. When considering the entire observation time, subcutaneous Interferon beta-1-a was the most frequently prescribed DMT (32.1%), followed by intramuscular Interferon beta-1-a (19.7%), Interferon beta-1-b (16.6%), Natalizumab (6.4%), Fingolimod (5.4%), Glatiramer acetate (5.4%), Teriflunomide (2.3%), Dimethyl Fumarate (2,1%), and no DMT (10.0%). Specifically, a limited number of patients, after starting the first prescribed DMT, spent at least part of the study without any DMT.

Overall annual healthcare costs were associated with age (Coef = -9.561; 95%CI = -17.072–2.051), and with baseline EDSS (Coef = 3.252; 95%CI = 1.706–4.797), but not with gender (p = 0.595), and with disease duration (Coef = -0.483; 95%CI = -3.350–2.384).

**Fig 2:**
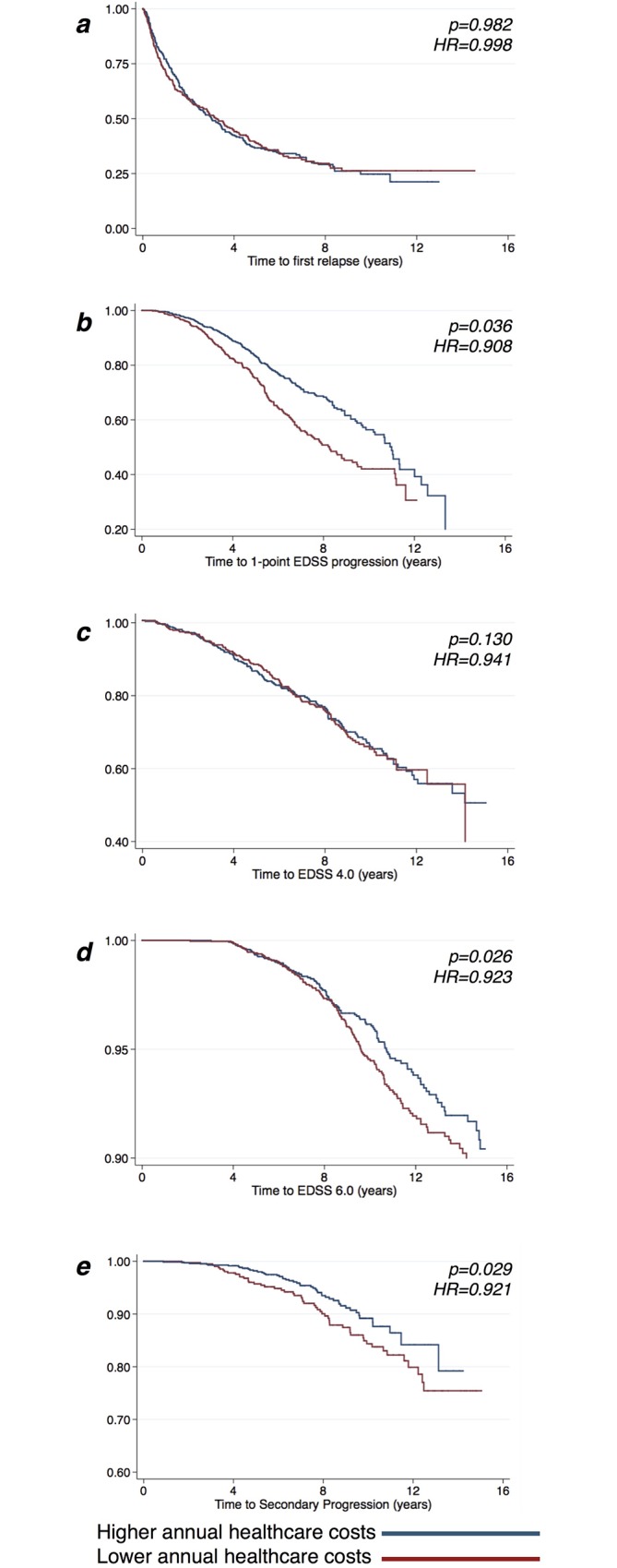
Kaplan-Meier curves for the probability of relapse occurrence, of 1-point EDSS progression, of reaching of EDSS 4.0, of reaching of EDSS 6.0, and of SP conversion, in relation to annual healthcare costs before the specific study endpoint was reached. Kaplan-Meier plots estimating the probability of relapse occurrence (**A**), of experiencing 1-point EDSS progression (**B**), of reaching of EDSS 4.0 (**C**), of reaching of EDSS 6.0 (**D**), and of SP conversion (**E**), in relation to the annual healthcare costs before the specific study endpoint was reached. P-values and hazard ratios (HR) are shown from time varying Cox regression models. For graphical purposes, healthcare costs have been categorized on the median value (the red line represents costs lower than the median value, whereas the blue line represents costs higher than the median value). EDSS: Expanded Disability Status Scale; HR: Hazard Ratio.

After adjusting for different covariates, 10% increase in the annual healthcare costs was associated with 1.1% reduction in the rate of 1-point EDSS progression (HR = 0.897; 95%CI = 0.820–0.981; Fig 2B), with 0.7% reduction in the reaching of EDSS 6.0 (HR = 0.925; 95%CI = 0.862–0.992; Fig 2D), and with 1.0% reduction in the conversion to SP (HR = 0.902; 95%CI = 0.838–0.971; Fig 2E), but not with the occurrence of the first relapse (HR = 0.993; 95%CI = 0.890–1.109; Fig 2A), and with the reaching of EDSS 4.0 (HR = 0.929; 95%CI = 0.859–1.005; Fig 2D) (Table 2).

**Table 2:** Healthcare costs for MS treatment and the rate of relapse occurrence, of 1-point EDSS progression, of reaching of EDSS 4.0, of reaching of EDSS 6.0, and of SP conversion. Healthcare costs for DMT administration and management before the specific endpoint was reached (considered as continuous variables), have been associated with the rate of relapse occurrence, of 1-point disability progression, of reaching of EDSS 4.0, of reaching of EDSS 6.0, and of SP conversion during the follow-up period. P-values, hazard ratios (HR), and 95% confidence intervals (95%CI) are shown from time varying Cox regression models, subsequently adjusted for age, gender, disease duration, and baseline EDSS.

	Unadjusted model				Adjusted model			
	*HR*	*95% CI*		*P-value*	*Adj*. *HR*	*95% CI*		*P-value*
		*Lower*	*Upper*			*Lower*	*Upper*	
**Relapse occurrence**	0.998	0.894	1.114	*0*.*982*	0.993	0.890	1.109	*0*.*913*
**1-point EDSS progression**	0.908	0.830	0.993	*0*.036*	0.897	0.820	0.981	*0*.018*
**Reaching of EDSS 4.0**	0.941	0.870	1.017	*0*.*130*	0.929	0.859	1.005	*0*.*070*
**Reaching of EDSS 6.0**	0.923	0.861	0.990	*0*.026*	0.925	0.862	0.992	*0*.030*
**SP conversion**	0.921	0.856	0.991	*0*.029*	0.902	0.838	0.971	*0*.006*

(*: p<0.05)

MS: Multiple Sclerosis; DMT: Disease Modifying Treatment; EDSS: Expanded Disability Status Scale; SP: secondary progression.

**Fig 3:**
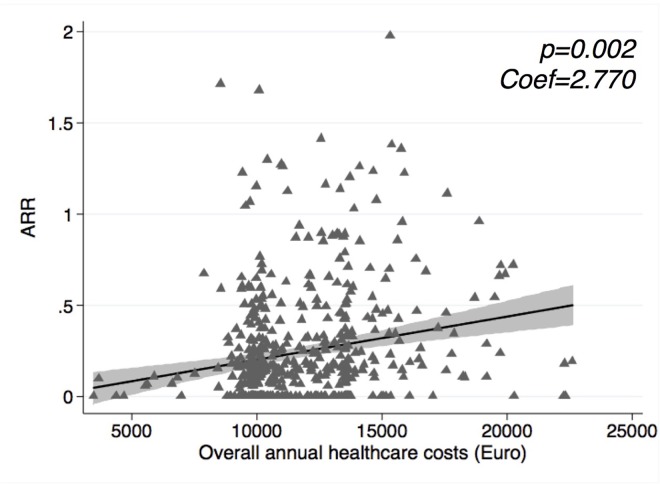
Scatter plot for overall annual healthcare costs and relapses. Scatter plot showing the relationship between overall annual healthcare costs and the ARR. P-value and coefficient from Poisson regression analysis are shown; 95% confidence intervals are represented in grey shadow. ARR: Annualised Relapse Rate; Coef: Coefficient.

Overall annual healthcare costs were positively associated with the ARR (Coef = 2.770; 95%CI = 1.056–4.483; and Coef = 2.468; 95%CI = 0.629–4.307 at the adjusted model) (Fig 3).

## Discussion

The present study found that higher healthcare costs for the use of more expensive DMTs from the early phases of MS, were associated with a lower risk of reaching milestones of short- (e.g. 1-point EDSS progression which occurred after 4–5 years from diagnosis, on average), and long-term disease evolution (e.g. reaching of EDSS 6.0 and conversion to SP, which took place after 8–10 years, on average). For instance, the use of treatments for highly active RRMS such as Fingolimod and Natalizumab, which are twice more expensive compared with Interferons [18], might have ideally halved the number of patients reaching EDSS 6.0 during 10 years in the present population (with a 5% overall reduction). Considering that previous studies have invariably found higher non-medical direct and indirect costs as disability progresses (i.e. rehabilitation, productivity loss) [8,31–34], it can be hypothesized that the delay or, ideally, the prevention of MS progression, also by using more expensive drugs, might determine long-term substantial rewards [2,33]. Noteworthy, in the present study, healthcare costs consisted of both the drug and the management procedures, in order to obtain a full evaluation of direct costs for treating MS, and were inflated to the most recent value to make cross-year comparisons possible.

There are different possible reasons underlying the association between healthcare costs for DMT administration and management, and MS progression. Healthcare costs in MS are driven by the use of DMTs, which are prescribed depending on the initial severity of MS and on its subsequent evolution. Indeed, the costs for DMTs are usually determined on the basis of their clinical effects as evaluated in clinical trials, and, so, they represent a surrogate marker of DMT efficacy. However, healthcare costs for DMT administration and management have never been tested in long-term real-life scenarios in relation to MS clinical outcomes. In previous observational studies trying to investigate the long-term efficacy of DMTs, Natalizumab determined more positive disease outcomes, compared with Interferon-beta or Glatiramer acetate, in a large propensity-matched cohort [28]. Similarly, the highest cumulative exposure (a parameter including both dosage and time) to Interferon beta-1a treatment had more beneficial effects on long-term outcomes, compared to the lowest, in patients with similar baseline clinical features [6]. Accordingly, the discontinuation of DMTs in patients with a more benign evolution determined a progressive disability accrual after 5 years, compared with subjects staying on continuous treatment with a more aggressive treatment approach [29]. Overall, clinical evidence support the effectiveness of highly-active, continuous and, subsequently, costly treatments seem in preventing or, at least, delaying MS progression. Accordingly, in the present study, patients who received more expensive DMTs, specifically indicated for a more aggressive disease evolution [16,17], presented better long-term outcomes, compared with subjects with relatively milder symptoms who received “low-cost” DMTs. This issue should be considered not only by physicians when profiling MS patients to prescribe the most suitable treatment [30], but also by policy makers when defining eligibility criteria for DMTs.

Among additional results, in the present cohort a higher relapse rate was associated with more expensive healthcare choices. Accordingly, previous studies showed that financial consequences of relapses depended not only on the temporary loss of productivity, but also on the subsequent change of treatment [35–37]. Indeed, overall annual healthcare costs, although not being associated with the risk of the first relapse, were positively correlated to the ARR. Conceivably, the first prescribed DMT was selected in consideration of baseline clinical features, and then changed in consideration of the subsequent disease evolution by escalating towards more expensive (and effective) treatments [18].

Limitations of the present study include the retrospective and non-randomized design, which, although generally considered acceptable for long-term studies in MS [23], might be responsible for a bias in the allocation to a first-line, second-line or no treatment. In addition, considering that in the present population the number of patients treated with highly-active treatments (e.g. Fingolimod and Natalizumab) was relatively low and DMTs more recently approved (e.g. Teriflunomide and Dimethyl Fumarate) were only marginally represented, a formal comparison among different DMTs was not performed. Moreover, the analysis did not include non-medical direct and indirect costs, and concomitant factors also affecting healthcare costs, such as comorbidities or presence of neutralising antibodies [37,38]. Finally, this is a single Centre experience and, so, different settings of care might have specific healthcare provision patterns [21].

In conclusion, the current landscape with newer and more expensive DMTs is a noticeable challenge for the economic performance of the healthcare systems. Hence, even if requiring short-term resource investments, a higher expenditure for treating MS has been associated with better disease evolution after 10 years. Current findings highlight the need for highly active and, possibly, expensive DMTs, in order to delay disease progression and its long-term burdensome economic consequences.
